# A Newly Synthesized Rhamnoside Derivative Alleviates Alzheimer's Amyloid-*β*-Induced Oxidative Stress, Mitochondrial Dysfunction, and Cell Senescence through Upregulating SIRT3

**DOI:** 10.1155/2020/7698560

**Published:** 2020-02-13

**Authors:** Yi Li, Jing Lu, Xin Cao, Hongwei Zhao, Longfei Gao, Peng Xia, Gang Pei

**Affiliations:** ^1^State Key Laboratory of Cell Biology, CAS Center for Excellence in Molecular Cell Science, Shanghai Institute of Biochemistry and Cell Biology, Chinese Academy of Sciences, University of Chinese Academy of Sciences, Shanghai, China; ^2^School of Life Science and Technology, ShanghaiTech University, Shanghai, China; ^3^Zhongshan Institute of Clinical Science, Fudan University, China; ^4^Chemical Biology Core Facility, Institute of Biochemistry and Cell Biology, Shanghai Institutes for Biological Sciences, Chinese Academy of Sciences, 200031 Shanghai, China; ^5^Shanghai EW Medicine Co. Ltd., China; ^6^Shanghai Key Laboratory of Signaling and Disease Research, Collaborative Innovation Center for Brain Science, School of Life Sciences and Technology, Tongji University, Shanghai, China; ^7^Institute for Stem Cell and Regeneration, Chinese Academy of Sciences, Beijing, China

## Abstract

Oxidative stress-induced mitochondrial dysfunction and cell senescence are considered critical contributors to Alzheimer's disease (AD), and oxidant/antioxidant imbalance has been a therapeutic target in AD. SIRT3 is a mitochondrial protein regulating metabolic enzyme activity by deacetylation and its downregulation is associated with AD pathology. In the present study, we showed that a newly synthesized rhamnoside derivative PL171 inhibited the generation of reactive oxidant species (ROS) induced by amyloid-*β*_42_ oligomers (A*β*_42_O), major AD pathological proteins. Moreover, the reduction of mitochondrial membrane potential (MMP) and the impairment of mitochondrial oxygen consumption triggered by A*β*_42_O were also prevented by PL171. Further experiments demonstrated that PL171 reduced the acetylation of mitochondrial proteins, and particularly the acetylation of manganese superoxide dismutase (MnSOD) and oligomycin-sensitivity-conferring protein (OSCP), two mitochondrial SIRT3 substrates, was suppressed by PL171. Mechanism studies revealed that PL171 upregulated SIRT3 and its upstream peroxisome proliferator-activated receptor-*γ* coactivator 1*α* (PGC-1*α*) under basal and A*β*_42_O-treated conditions. The inhibition of SIRT3 activity could eliminate the protective effects of PL171. Further, long-term treatment with A*β*_42_O increased the number of senescent neuronal cell, which was also alleviated by PL171 in a SIRT3-dependent manner. Taken together, our results indicated that PL171 rescued A*β*_42_O-induced oxidative stress, mitochondrial dysfunction, and cell senescence via upregulating SIRT3 and might be a potential drug candidate against AD.

## 1. Introduction

The neuropathological hallmark of Alzheimer's disease (AD) is the deposition of extracellular amyloid plaques in the brain due to the imbalance in the production and clearance of amyloid-*β* (A*β*) [1], as well as intracellular neurofibrillary tangles [2], leading to the damage and death in neurons. A*β* secreted outside exists in different assembly states, and a series of evidences demonstrated that soluble A*β* oligomers are more pathogenic than larger, insoluble, highly aggregated fibril [3, 4]. Mitochondria are dynamic organelles in eukaryotic cells playing a central role in ATP production, cellular calcium buffering, and apoptosis [5]. The reduction of mitochondria in its mass and function has emerged as another pathological feature in AD [6]. In recent years, some studies have shown that A*β* is imported into the mitochondria via the translocase of the outer membrane complex, providing a strong rationale that mitochondria also serve as targets for A*β*, contributing to cognitive decline and memory loss [7, 8]. A*β* destroyed mitochondrial homeostasis and interfered with the enzymatic activity of the complex in the mitochondrial electron transport chain (ETC), resulting in the impairment of the mitochondrial membrane potential (MMP) [9, 10]. And A*β* could cause serious oxidative damage by the overproduction of reactive oxidative species (ROS) and damage mitochondrial oxygen consumption directly leading to the reduction of ATP production [11].

Protein acetylation is a posttranslational process regulating global mitochondrial functions [12]. SIRT3 belongs to the sirtuin family and is located in the mitochondrial matrix, exhibiting a robust deacetylase activity [13, 14]. It regulates the activity of mitochondrial metabolic enzymes, such as manganese superoxide dismutase (MnSOD) [15] and oligomycin-sensitivity-conferring protein (OSCP) [16], by deacetylation and thereby reducing the overproduction of ROS under oxidative stress-dependent conditions such as aging and neural degeneration [13]. It has been found that SIRT3 is downregulated in the brain of AD patients and analyzing SIRT3 level may contribute to AD diagnosis [17, 18]. Therefore, promoting SIRT3 expression or function could be a promising therapeutic strategy for AD treatment. Rhamnose and rhamnoside have antioxidant effects [19, 20], while whether they could prevent A*β*-induced neuron dysfunction is unknown. The present study demonstrated that a newly synthesized rhamnoside derivative PL171 attenuated A*β*_42_ oligomer- (A*β*_42_O-) induced oxidative stress, mitochondrial dysfunction, and cell senescence by upregulating SIRT3-mediated antioxidant effects, indicating PL171 can counteract A*β*_42_O defects via SIRT3.

## 2. Material and Methods

### 2.1. The Synthesis of PL171

The (4-*O*-*tert*-butyldimethylsilyl) ferulic chloride [21] (653 mg, 2 mmol) in dry CH_2_Cl_2_ 5.0 mL was added to the solution of 2,3,4-tri-*O*-acetyl-*α*-*L*-rhamnopyranosylamine [22] (550 mg, 1.9 mmol) in a flask with the mixture of dry CH_2_Cl_2_ 10 mL and pyridine 0.1 mL at 0°C. The resulting mixture was stirred for 2 hours at rt, then diluted with CH_2_Cl_2_ 20 mL. The mixture was washed with brine and the combined organic layers were dried over MgSO_4_ and concentrated in vacuum. The residue was purified by chromatography on silica gel with eluent (petroleum ether-EtOAc, 10 : 1 to 2 : 1), and the *β*-L-rhamnopyranoside product 820 mg (75%) and *α*-L-rhamnopyranoside product 120 mg (11%) were obtained, respectively.

Then, the *β*-L-rhamnopyranoside product (500 mg, 0.86 mmol) was dissolved in 8.0 mL of dry THF, and TBAF (1 M in THF, 0.86 mL, 0.86 mmol) was added dropwise to the solution at rt. The reaction mixture was stirred for 2 hours, after which it was diluted with THF 20 mL. The mixture was washed with brine and the combined organic layers were dried over MgSO_4_, and concentrated in vacuum. The residue was purified by chromatography on silica gel with eluent (petroleum ether-EtOAc, 5 : 1 to 1 : 1), and the phenol product 340 mg (0.73 mmol, 85%) was obtained.

The obtained phenol product 300 mg (0.65 mmol) was dissolved in 3.0 mL of CH_2_Cl_2_. 33% CH_3_NH_2_ in CH_3_OH 0.5 mL was added to the solution at 0°C during 5 min. The resulting mixture was stirred for 1 hour at 0°C. The mixture was concentrated in vacuum and the resulting residue was purified by chromatography on silica gel with eluent (CH_2_Cl_2_-CH_3_OH, 20: 1 to 6: 1), and 140 mg (0.41 mmol, 64%) of PL171 was obtained. ESI (+)-MS: 340.3 (M+1)^+^; H-NMR (CD_3_OD, 400 MHz) *δ* 7.56 (d, *J* = 12 Hz, 1H) 7.19 (d, *J* = 4 Hz, 1H), 7.07 (dd, *J*_1_ = 6 Hz, *J*_2_ = 1 Hz, 1H), 6.82 (d, *J* = 4 Hz, 1H), 6.62 (d, *J* = 12 Hz, 1H), 5.28 (d, *J* = 1 Hz, 1H), 3.92 (s, 3H), 3.85 (d, *J* = 4 Hz, 1H), 3.54-3.52 (m, 1H), 3.38-3.34 (m, 2H), 1.32 (d, *J* = 4 Hz, 3H). ^13^C NMR (CD_3_OD, 400 MHz) *δ* 168.58, 149.92, 149.10, 143.64, 127.92, 123.51, 118.04, 116.32, 111.55, 79.06, 75.29, 75.22, 73.41, 72.30, 56.28, 17.92.

A flowchart to summarize the synthesis of PL171 is provided in Fig. [Supplementary-material supplementary-material-1]. The ^1^H-NMR and ^1^H-^1^H COSY spectra are provided in Figs. [Supplementary-material supplementary-material-1] and [Supplementary-material supplementary-material-1].

### 2.2. A*β*_42_ Oligomers Preparation

The A*β*_42_O were prepared according to the previous publications with some modifications [23–25]. A*β*_42_ peptides were purchased from (GenicBio, A-42-T-2). Briefly, the hexafluoroisopropanol- (HFIP-) treated A*β*_42_ peptides were resuspended in dimethyl sulfoxide (DMSO) and then diluted in DMEM/F12 phenol-red free medium to achieve a 100 *μ*M concentration. The diluted A*β*_42_ peptides were then vortexed for 15 s followed by incubation for 24 h at 4°C. The formation of A*β*_42_O were previously validated in our laboratory by dot blots, atomic force electromicroscopy, and western blot assays [23, 26].

### 2.3. Cell Culture

SK-N-SH cells were purchased from ATCC. The cell line was cultured in Modified Eagle's Medium (MEM) with 10% fetal bovine serum (FBS) and 100 U/mL penicillin and 0.1 mg/mL streptomycin in a humidified incubator with 5% CO2/95% air (*v*/*v*) at 37°C.

### 2.4. Cell Viability

SK-N-SH cells were seeded in 96-well plate at 1 × 10^4^ cells/well. After the treatment with PL171 for 24 h at indicated concentrations, cell viability was detected using Cell Titer-Glo Luminescent Assay (Promega, G7573), following the manufacturer's guidelines. The values were measured by BioTek SynergyNEO (BioTek, USA).

### 2.5. Mitochondria Isolation

The cellular mitochondria were extracted following the manufacturer's instructions with some modifications (Beyotime, C3601). SK-N-SH cells (1.5 × 10^6^) were seeded into 60 mm dishes. After the required treatments, cells were washed once with PBS, dissociated with trypsin-EDTA solution, and collected by centrifugation at 200 g for 10 min. The cell pellets were gently resuspended in PBS precooled in an ice bath followed by centrifugation at 600 g for 5 min at 4°C. The pellets were gently resuspended with 1 mL mitochondrial separation reagent supplemented with (100 *μ*M) PMSF and then incubated on ice for 10 minutes. Cell suspensions were then homogenized on ice with a 1 cc insulin syringe 28G1/2, drawing through the needle 10 times. After centrifugation at 600 g for 10 min at 4°C, the supernatants were collected and recentrifuged at 11,000 g for 10 min at 4°C to get the mitochondria. The mitochondria lysates were then used for western blot analysis.

### 2.6. ROS Assay

2′,7′-Dichlorodihydrofluorescein diacetate (DCFH-DA) (Beyotime, S0033) was used as a probe to detect intracellular ROS levels. Briefly, SK-N-SH cells were seeded in a 96-well plate at 1 × 10^4^ cells/well density. Cells were treated with A*β*_42_O or PL171 at indicated concentrations followed by staining with 10 *μ*M DCFH-DA in the serum and phenol-red free medium for 30 min at 37°C in a humidified incubator with 5% CO_2_/95% air (*v*/*v*) at 37°C. The cells were washed with PBS twice and then observed under a laser-scanning confocal microscope (Operetta, Perkin Eimer, USA). Alternatively, the cells in the 96-well black plate were extracted with 1% Triton X-100 for 10 min at 37°C and the fluorescence was measured using BioTek SynergyNEO (BioTek, USA) at an excitation wavelength of 488 nm and an emission wavelength of 525 nm.

#### 2.6.1. Mitochondrial ROS Detection

Cells were treated with 10 *μ*M A*β*_42_O for 24 h following preincubation with or without 30 *μ*M PL171 for 4 h. At the end of treatment, cells were costained with 2.5 *μ*M MitoSOX Red mitochondrial superoxide indicator (Invitrogen, M36008) and 3 *μ*g/mL the nuclear staining dye Hoechst (Beyotime, C1022) for 20 min at 37°C. The fluorescent signals were recorded using BioTek SynergyNEO at 510/580 nm (excitation/emission) for MitoSOX and 350/461 nm for Hoechst. The MitoSOX fluorescence signal was normalized to the Hoechst.

### 2.7. Measurement of Mitochondrial Membrane Potential

SK-N-SH cells were seeded into a 96-well plate (Costar, 3904) at the density of 1 × 10^4^ cells/well. Cells were challenged of A*β*_42_O or A*β*_42-1_O at indicated concentrations or pretreated with PL171 for 4 or 24 h followed by incubation with 10 *μ*M A*β*_42_O for another 24 h. JC-1 kits (Beyotime, C2006) were used to assess the MMP level of cells according to the manufacture's protocols. Briefly, cells were loaded with mixed JC-1 staining solution for 30 minutes at 37°C and then washed twice with the staining buffer. The cells were observed under a Zeiss Observer Z1 microscope. The fluorescence intensity was detected using the BioTek SynergyNEO (BioTek, USA) at 490/530 nm (green) for monomers and 525/590 nm for aggregates (red), and the membrane potential was represented as the ratio of red/green fluorescence intensity.

### 2.8. Oxygen Consumption Rate Measurement

The cellular oxygen consumption rate (OCR) of SK-N-SH cells was examined using SeahorseXFe24 following the manufacturer's guidance (Seahorse Bioscience). SK-N-SH cells were seeded in XF24-well microplates at 3 × 10^4^ cells/well. The cells were pretreated with or without 30 *μ*M of PL171 for 4 h followed by stimulation with 10 *μ*M A*β*_42_O for 24 h. The cells were then incubated in a nonbuffered bicarbonate-free DMEM (Sigma) containing 25 mM glucose, 2 mM glutamax, and 1 mM sodium pyruvate at 37°C in an incubator without CO_2_ for 45 min. OCR was measured under basal condition and also after the injection of oligomycin (1 *μ*M), FCCP (1 *μ*M), rotenone (1 *μ*M), and antimycin A (1 *μ*M). Data were analyzed using Seahorse XF-24 software.

### 2.9. SA-*β*-Gal Assay

Cell senescence was measured by SA-*β*-gal staining using a commercial kit (Beyotime, C0602). SK-N-SH cells (5 × 10^4^ cells/well) were cultured in the medium with 5% FBS in 24-well plates. After 72 h treatments with A*β*_42_O in the absence or presence of PL171, cells were prepared for SA-*β*-gal staining following the manufacturer's guidelines. The cells were imaged by a Zeiss Observer Z1 microscope. The blue-stained cells from at least 10 different fields (60-100 cells/field) were counted under each experiment.

### 2.10. Reverse Transcription and Quantitative Real-Time PCR

After treatment with PL171 at indicated concentrations, cells at 2 × 10^5^ cells/well density were extracted by 1 mL TRI Reagent (Sigma, T9424) to obtain total RNA according to the manufacturer's instructions. Reverse transcription was conducted using PrimeScript RT master mix (TaKaRa, RR036B) under the following conditions: 37°C, 15 min and 85°C, 15 sec. Then, the reaction consists of 4 *μ*L of prediluted cDNA in a total volume of 25 *μ*L reaction containing 0.25 *μ*M each primer. And gene transcripts were analyzed by quantitative real-time PCR conducted with 2x HotStart SYBR Green qPCR master mix (ExCell Bio, MB000-3013) on a Stratagene Mx3000P (Agilent Technologies). The reaction parameters were as follows: 95°C for 10 min; 95°C for 30 s, 40 cycles; 60°C for 30 s; 72°C for 30 s. An additional cycle was performed for evaluation of primer's dissociation curve: 95°C for 1 min, 60°C for 30 s and 95°C for 30 s. Each cDNA sample was amplified twice. HPRT was used as an internal control. Primers used were as follows:

SIRT1, forward 5′-AAGTTGACTGTGAAGCTGTACG-3′, reverse 5′-TGCTACTGGTCTTACTTTGAGGG-3′; SIRT3, forward 5′-CCCCAAGCCCTTTTTCACTTT-3′, reverse 5′-CGACACTCTCTCAAGCCCA-3′; PGC-1*α*, forward 5′TCTGAGTCTGTATGGAGTGACAT-3′, reverse 5′-CCAAGTCGTTCACATCTAGTTCA-3′; HPRT, forward 5′-CCTGGCGTCGTGATTAGTGAT-3′, reverse 5′-AGACGTTCAGTCCTGTCCATAA-3′.

### 2.11. Western Blot

Cells (1 × 10^5^ cells/well) were treated with PL171 for 24 h or pretreated with PL171 for 4 h followed by A*β*_42_O treatment for another 24 h. For mitochondria lysate preparation, cells at 1.5 × 10^6^ cells/well density were seeded and mitochondria were isolated as previously described. Total cell lysates or mitochondria lysates were separated by 10 or 12% sodium dodecyl sulfate-polyacrylamide gel electrophoresis (SDS-PAGE) and transferred onto nitrocellulose membranes (400 mA constant current, 2 h, 4°C). Membranes were blocked with 5% nonfat milk in TBS containing 0.1% Tween-20 for 1 h at room temperature (RT). Membranes were subsequently incubated with relevant antibodies: SIRT3 (1 : 1000, Cell Signaling Technology, 5490S), ATP5A (1 : 1000, Abclonal, A5884), SIRT1 (1 : 1000, Proteintech, 13161-1-AP), OSCP (1 : 500, Santa Cruz Biotechnology, sc-365162), ATP5Ok139 (1 : 200, Abcam, ab214339), SOD2 (1 : 500, Santa Cruz Biotechnology, sc-133134), SOD2k68 (1 : 1000, Abcam, ab137037), PGC-1*α* (1 : 1000, Proteintech, 66369-1-Ig), AMPK*α* Rabbit Monoclonal Antibody (1 : 1000, Beyotime, AF1627), Phospho-AMPK*α* (Thr172) Antibody (1 : 1000, Beyotime, AA393), and actin (1 : 1000, Sigma, #A2066), at 4°C overnight followed by horseradish peroxidase- (HRP-) conjugated secondary antibody. Membranes were then incubated with an ECL substrate and visualized by Mini Chemiluminescent Imaging and Analysis System.

### 2.12. Statistical Analysis

The data were analyzed by Prism 6.0 (GraphPad Software Inc., San Diego, CA). Unpaired Student's *t*-test (two-tailed) was applied for the comparisons of two datasets, and one-way or two-way analysis of variance (ANOVA) with Bonferroni's posttest was used where more than two datasets were compared. Statistical significance was accepted at *p* < 0.05.

## 3. Results

### 3.1. PL171 Dose Dependently Inhibited A*β*_42_O-Induced ROS Production in SK-N-SH Cells

**Figure 1:**
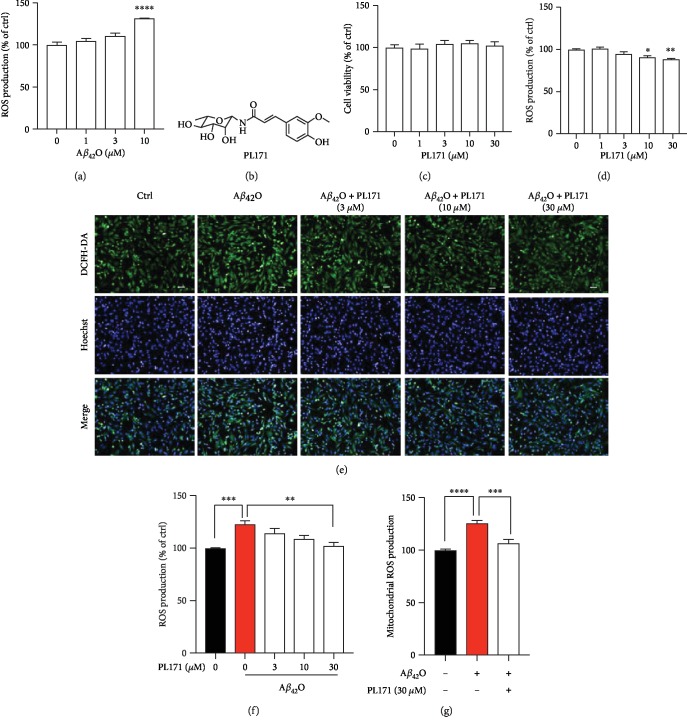
PL171 dose dependently inhibited A*β*_42_O-induced ROS production in SK-N-SH cells. (a) ROS generation in SK-N-SH cells incubated with A*β*_42_O (1-10 *μ*M) for 24 h and then stained with DCFH-DA. The data were normalized to the control. (b) The chemical structure of PL171. (c) Cells were treated with PL171 (1-30 *μ*M) for 24 h and cell viability was measured by CellTiter-Glo Assay. (d) The ROS generation of SK-N-SH cells treated with PL171 (1-30 *μ*M) for 24 h followed by staining with DCFH-DA dye. (e) The representative image of SK-N-SH cells preincubated with PL171 (3-30 *μ*M) for 4 h, treated with A*β*_42_O (10 *μ*M) for another 24 h, and then costained with DCFH-DA and Hoechst. The pictures were obtained by Operetta. Scale bars, 50 *μ*m. (f) The quantification of (e), showing relative ROS generation of cells pretreated with PL171 for 4 h before A*β*_42_O (10 *μ*M) stimulation for 24 h. (g) Mitochondrial ROS production in the cells challenged of A*β*_42_O (10 *μ*M, 24 h) with or without PL171 (30 *μ*M, 4 h) preincubation. The signal of MitoSOX was normalized to Hoechst. The data are presented as mean ± SEM, *n* ≥ 3 independent experiments, ^∗^*p* < 0.05, ^∗∗^*p* < 0.01, ^∗∗∗^*p* < 0.001, and ^∗∗∗∗^*p* < 0.0001, analyzed by one-way ANOVA followed by Bonferroni's test.

A*β*_42_O could induce the generation of ROS, thus causing oxidative stress of neurons. Here, we treated human neuronal cells SK-N-SH with different concentrations of A*β*_42_O for 24 h and measured the cellular ROS level by staining with DCFH-DA, which has no fluorescence and can pass freely through plasma membrane, and produce fluorescent DCF when oxidized by ROS. Data showed that treatment with A*β*_42_O dose dependently increased DCF fluorescence and A*β*_42_O at 10 *μ*M significantly promoted the signal by about 30% to the control (Figure 1(a)]. Thus, 10 *μ*M of A*β*_42_O was applied for the subsequent experiments. We then tested whether PL171, as shown in the structure (Figure 1(b)), can modulate A*β*_42_O-induced ROS promotion. Treatment with PL171 up to 30 *μ*M for 24 h did not influence cell viability (Figure 1(c)). The effect of PL171 on the basal level of ROS was investigated. We observed that after 24 h treatment, PL171 decreased basal ROS production in a dosage-dependent manner with around 14% reduction made by 30 *μ*M of PL171 (Figure 1(d)). A*β*_42_O (10 *μ*M) consistently induced the increase of ROS which however was dose dependently reduced by the pretreatment with PL171 (Figures 1(e) and 1(f)). PL171 at 30 *μ*M almost completely inhibited A*β*_42_O-induced ROS generation. To specifically detect the mitochondrial ROS, a mitochondrial superoxide indicator, MitoSOX, was applied. Data consistently showed that A*β*_42_O (10 *μ*M, 24 h) stimulated mitochondrial ROS by about 26% which was significantly suppressed by the preincubation with PL171 (30 *μ*M, 4 h) (Figure 1(g)). These results indicate that PL171 protects neuronal cells from A*β*_42_O-induced oxidative stress.

### 3.2. PL171 Prevented A*β*_42_O-Induced MMP Reduction in SK-N-SH Cells

**Figure 2:**
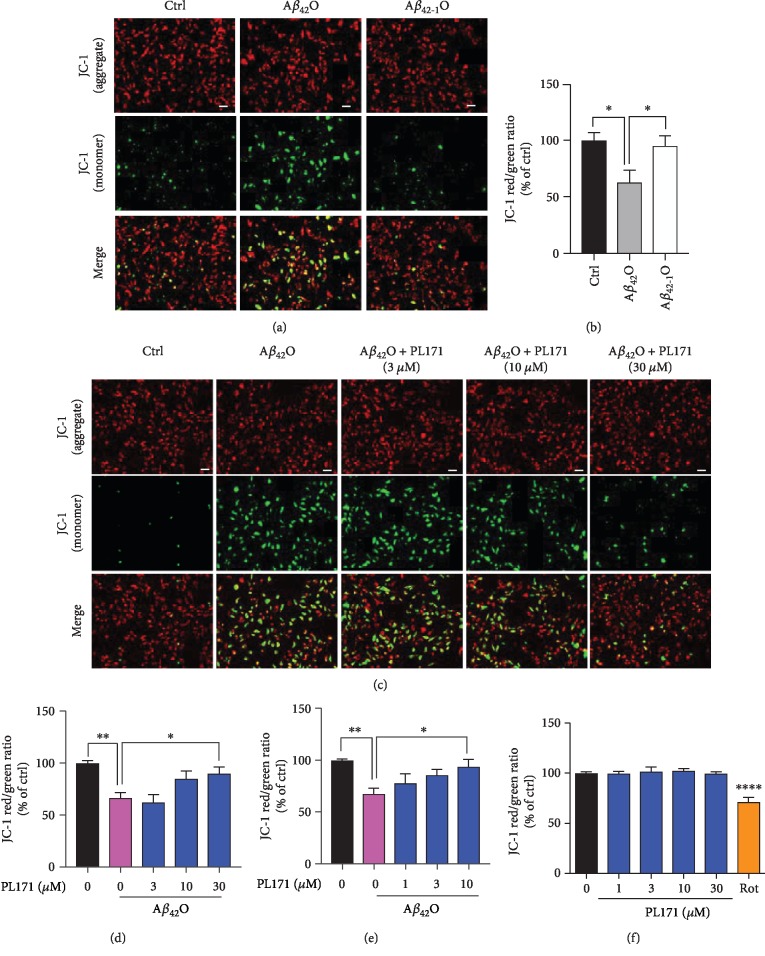
PL171 prevented A*β*_42_O-induced MMP reduction in SK-N-SH cells. (a) The representative MMP images of SK-N-SH cells incubated with A*β*_42_O or A*β*_42-1_O (10 *μ*M) for 24 h and then stained with JC-1 dye. Green (excitation: 490; emission: 530); red (excitation: 525; emission: 590). Scale bars, 50 *μ*m. (b) The ratio of red/green fluorescence from (a). (c) The representative images of SK-N-SH cells preincubated with PL171 for 4 h followed by treatment with A*β*_42_O (10 *μ*M) for 24 h. Cells were then stained with JC-1 dye and imaged by Zeiss Observer Z1 microscope. (d) The fluorescence intensity in (c) was quantified using BioTek SynergyNEO. Scale bars, 50 *μ*m. (e) Cells were treated as (d) but with PL171 preincubation for 24 h. (f) Cells were treated with PL171 (1-30 *μ*M) for 24 h, stained with JC-1 dye, and detected by BioTek SynergyNEO. Rotenone (Rot) was the positive control. The data are presented as mean ± SEM, *n* ≥ 3 independent experiments, ^∗^*p* < 0.05, ^∗∗^*p* < 0.01, and ^∗∗∗∗^*p* < 0.0001, analyzed by one-way ANOVA followed by Bonferroni's test.

A*β*_42_O can induce the loss of MMP. In the present study, JC-1 probe was used to evaluate MMP in SK-N-SH cells. Red fluorescence and green fluorescence represented high and low mitochondrial membrane permeability, respectively, and the ratio could represent the change of MMP. Compared with the control group, treatment with A*β*_42_O largely enhanced green fluorescence intensity (Figure 2(a)) and significantly reduced the red/green fluorescence (Figure 2(b)), indicating MMP depolarization induced by A*β*_42_O. By contrast, A*β*_42-1_ as the negative control had no obvious effect (Figures 2(a) and 2(b)). The effect of A*β*_42_O on MMP was time and dosage dependent. A*β*_42_O (10 *μ*M) decreased MMP by about 24%, 32%, and 36% for 8 h, 16 h, and 24 h, respectively (Fig. [Supplementary-material supplementary-material-1]). Interestingly, pretreatment with PL171 for 4 h dose dependently prevented A*β*_42_O-impaired MMP (Figures 2(c) and 2(d)). A*β*_42_O (10 *μ*M, 24 h) induced the reduction of MMP by 34% which was attenuated to around 10% by preincubation with 30 *μ*M of PL171 for 4 h. This protective effect of PL171 was even more profound when extending the period of PL171 preincubation to 24 h (Figure 2(e)). And meanwhile, PL171 did not change MMP in the cells without A*β*_42_O while rotenone as a positive control produced around 37% reduction (Figure 2(f)).

### 3.3. PL171 Inhibited A*β*_42_O-Induced Reduction of Oxygen Consumption in SK-N-SH Cells

**Figure 3:**
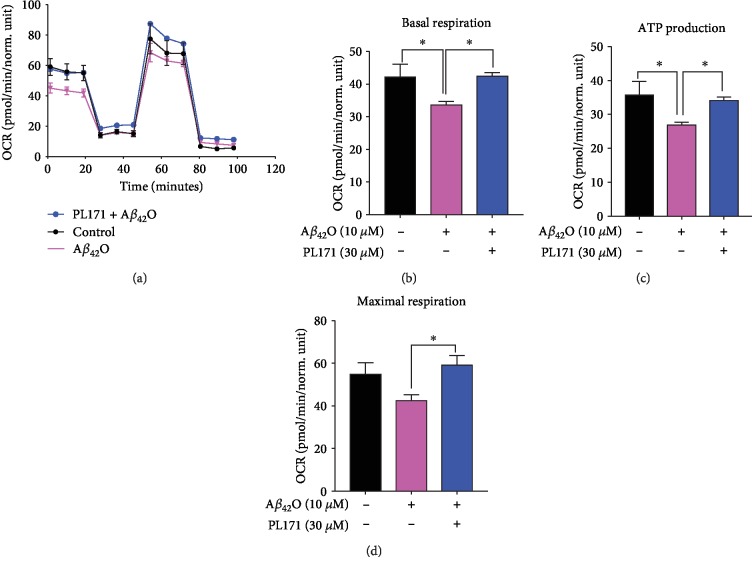
PL171 inhibited A*β*_42_O-induced reduction of oxygen consumption in SK-N-SH cells. Seahorse assays showing mitochondrial bioenergetics in SK-N-SH cells preincubated without or with PL171 (30 *μ*M) for 4 h followed by stimulation with A*β*_42_O (10 *μ*M) for 24 h. (a) The representative graph of the mitochondrial stress test detailing the four key parameters of mitochondrial function through sequential addition of oligomycin (1 *μ*M), FCCP (1 *μ*M), and rotenone/antimycin A (1 *μ*M each), which allowed the measurement of basal respiration (b), mitochondrial ATP production (c), and the maximal respiration (d). The data are presented as mean ± SEM, *n* = 3 independent experiments, ^∗^*p* < 0.05, analyzed by one-way ANOVA followed by Bonferroni's test.

Previous results showed that A*β* accumulated in the mitochondria, thus resulting in ATP depletion, decline of respiration rate, and low respiratory enzyme activity [10, 27]. To further detect the effect of PL171 on mitochondrial function, we analyzed oxygen consumption rate (OCR) using a Seahorse instrument. In our study, compared to the control group, A*β*_42_O (10 *μ*M, 24 h) impaired OCR, and however, the presence of PL171 (30 *μ*M, 4 h pretreatment) inhibited A*β*_42_O-induced mitochondrial impairment (Figure 3(a)). A*β*_42_O declined basal respiration by 21% which was rescued to the control level by preincubation with 30 *μ*M of PL171 for 4 h (Figure 3(b)). Meanwhile, A*β*_42_O reduced ATP production by about 25% while pretreatment with PL171 (30 *μ*M) for 4 h restored the ATP level to the level similar as the control (Figure 3(c)). Compared with the control group, A*β*_42_O impaired the mitochondrial maximal respiration by 22% which was also prevented in the presence of PL171 completely (Figure 3(d)). Taken together, our data suggest that PL171 can inhibit A*β*_42_O-induced reduction of oxygen consumption, including ATP production, basal respiration, and maximal respiration and maintain healthy mitochondrial function.

### 3.4. PL171 Promoted Mitochondrial SIRT3 Level and Its Activity

**Figure 4:**
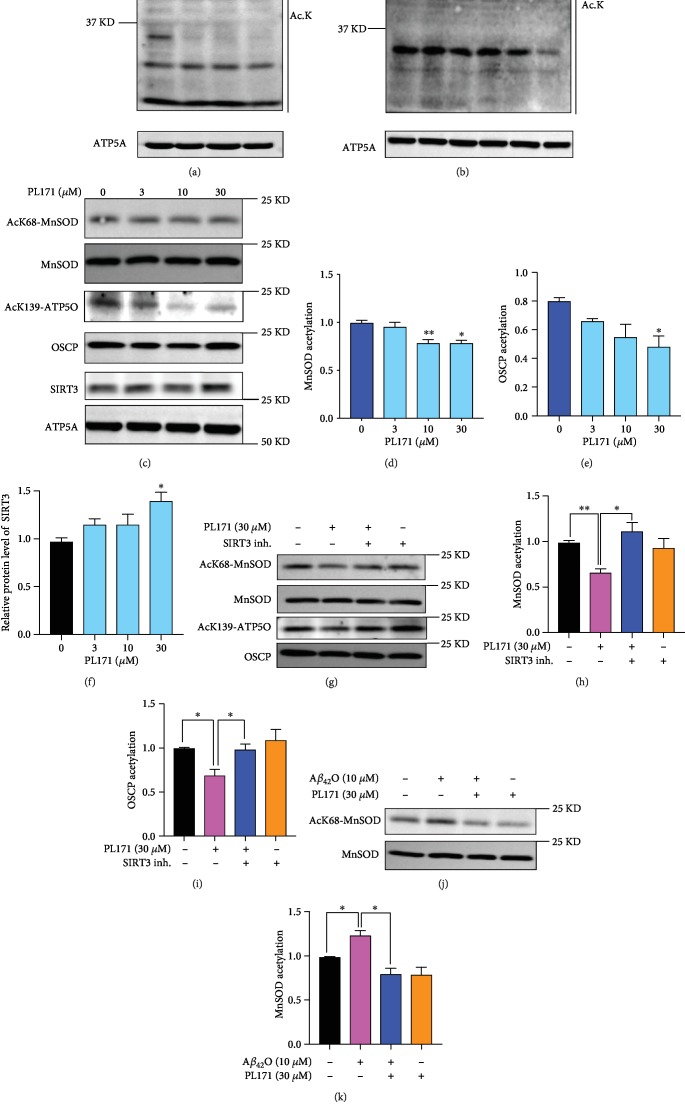
PL171 promoted SIRT3 levels and its activity. (a) SK-N-SH cells were incubated with PL171 at different dosages as indicated for 24 h. Mitochondria were isolated and the lysates were prepared for the measurement of total acetylation of mitochondrial proteins using the anti-acetylation antibody (Ac-K) by western blotting. ATP5A was used as a loading control. (b) SK-N-SH cells were treated with 30 *μ*M PL171 at different time points as indicated. Mitochondrial lysates were prepared for the detection of mitochondrial protein acetylation using the anti-acetylation antibody (Ac-K) by western blotting. ATP5A was used as a loading control. (c) After the incubation with PL171 at indicated concentrations for 24 h, mitochondrial lysates were prepared and analyzed using western blotting with indicated antibodies. (d–f) Quantification of relative acetylated MnSOD (AcK68-MnSOD/MnSOD, d), relative acetylated OSCP (AcK139-OSCP/OSCP, e), and relative SIRT3 level (SIRT3/ATP5A, f). (g) Representative blot showing the acetylation of MnSOD and OSCP in the mitochondrial lysates of SK-N-SH cells, challenged of PL171 (30 *μ*M) with or without SIRT3 inhibitor (3-TYP, 20 *μ*M) for 24 h. (h) Quantification of relative MnSOD acetylation in (g). (i) Quantification of relative OSCP acetylation in (g). (j) Representative blot presenting the acetylation of MnSOD in the mitochondrial lysates of SK-N-SH cells pretreated with 30 *μ*M PL171 for 4 h followed by stimulation with 10 *μ*M A*β*_42_O for 24 h. (k) Quantification of relative MnSOD acetylation in (j). The data are presented as mean ± SEM, *n* ≥ 3 independent experiments, ^∗^*p* < 0.05, ^∗∗^*p* < 0.01, analyzed by one-way ANOVA followed by Bonferroni's test.

Mitochondrial protein acetylation is tightly associated with mitochondrial function [28, 29]. Firstly, we detected the effect of PL171 on the acetylation status of mitochondrial proteins. SK-N-SH cells were treated with various concentrations of PL171 for 24 h followed by mitochondria isolation. Data showed that PL171 reduced total acetylation of mitochondrial protein dose dependently (Figure 4(a)). To investigate the time course of mitochondrial protein deacetylation, cells were treated with PL171 at 30 *μ*M for 0.5-24 h, and data presented that 24 h treatment produced maximum reduction of acetylation (Figure 4(b)). Since SIRT3 plays a significant role in mitochondrial protein deacetylation [30], the expression of SIRT3 in mitochondria was determined. The immunoblotting showed that PL171 increased mitochondrial SIRT3 by 36% (Figures 4(c) and 4(f)). Furthermore, we asked if the upregulation of SIRT3 promoted its activity for substrate deacetylation. The acetylation level of the SIRT3 substrates, manganese superoxide dismutase (SOD2) and oligomycin-sensitivity-conferring protein (OSCP), was detected using antibodies that specifically detect MnSOD acetylation at K-68 and OSCP acetylation at K-139 by immunoblotting. PL171 decreased the acetylation of MnSOD and OSCP in a dose-dependent manner and 30 *μ*M of PL171 reduced acetylation of MnSOD (SODk68/MnSOD) and OSCP (ATP5O/OSCP) by about 20% and 36%, respectively (Figures 4(c)–4(e)). However, treatment with a SIRT3 inhibitor (3-TYP, 20 *μ*M) significantly blocked the effect of PL171 (Figures 4(g)–4(h)). Furthermore, A*β*_42_O (10 *μ*M) increased the acetylation level of MnSOD which was significantly downregulated by preincubation with 30 *μ*M of PL171 for 4 h (Figures 4(j) and 4(k)). All these demonstrate that PL171 can protect mitochondrial function by facilitating mitochondrial protein deacetylation through promoting SIRT3 function.

### 3.5. PL171 Restored SIRT3 and PGC-1*α* Reduction Induced by A*β*_42_O

**Figure 5:**
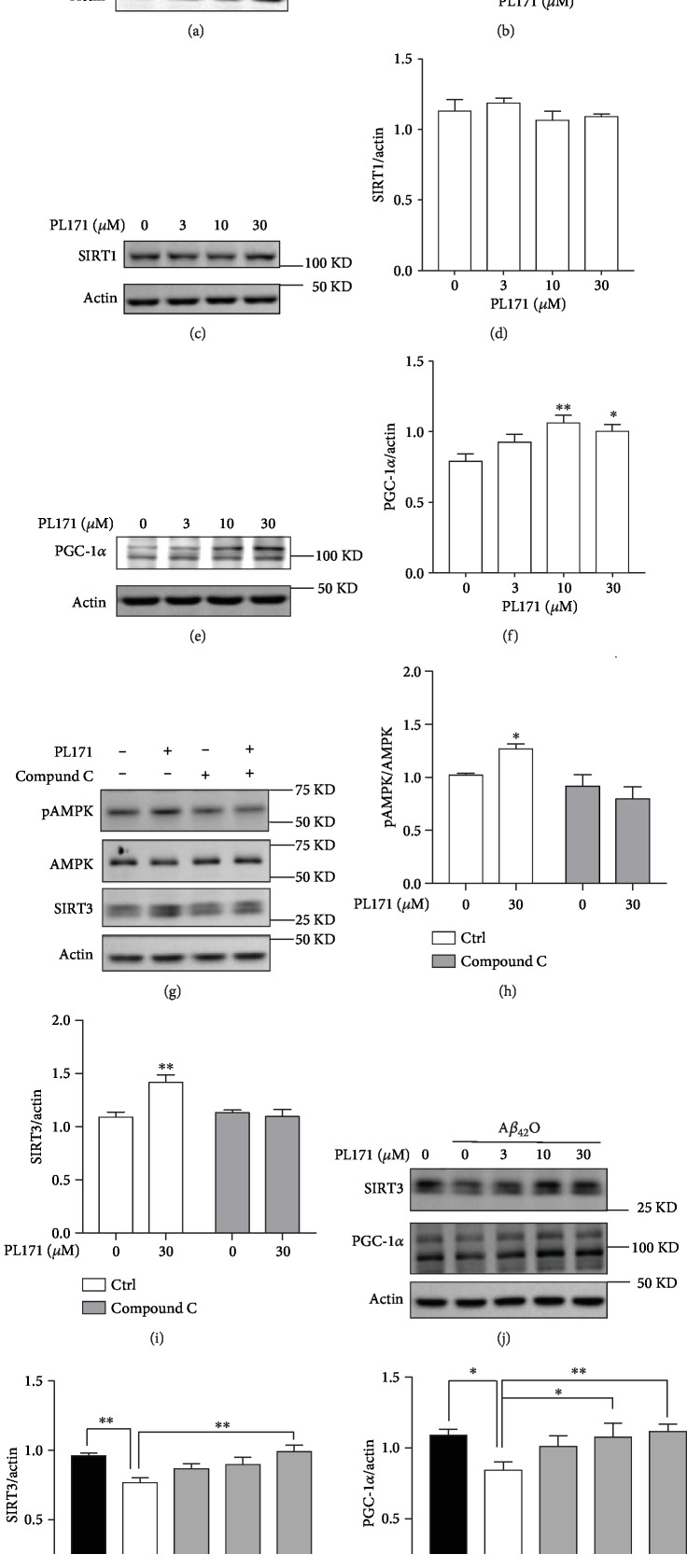
PL171 restored cellular SIRT3 and PGC-1*α* reduction induced by A*β*_42_O. (a) Cells were treated with PL171 at different concentrations, and cell lysates were prepared and analyzed using western blotting with SIRT3 antibody. Actin was used as a loading control. (b) The quantification of relative SIRT3 protein level in (a). (c) The cell lysates in (a) were analyzed against SIRT1 antibody. (d) The quantification of relative SIRT1 protein level in (c). (e) The cell lysates in (a) were analyzed against PGC-1*α* antibody. (f) The quantification of relative PGC-1*α* protein level in (e). (g) Cells were pretreated with or without compound C (3 *μ*M, 30 min) followed by stimulation with PL171 (30 *μ*M, 24 h). The protein expression of AMPK phosphorylation (pAMPK), total AMPK, and SIRT3 was detected by western blotting. Actin was used as a loading control. The relative pAMPK and SIRT3 were quantified (h, i). (j) After preincubation with PL171 as indicated, cells were treated with 10 *μ*M A*β*_42_O for 24 h, and then cell lysates were prepared and analyzed using western blotting against SIRT3 and PGC-1*α* antibody. (k, l) The quantifications of relative SIRT3 and PGC-1*α* protein level in (j). The data are presented as mean ± SEM, *n* ≥ 3 independent experiments, ^∗^*p* < 0.05, ^∗∗^*p* < 0.01, analyzed by one-way or two-way ANOVA followed by Bonferroni's test.

We then further explored the mechanism by which PL171 prevents A*β*_42_O-induced mitochondrial dysfunction. Consistently, PL171 dose dependently enhanced the expression of SIRT3 in total cell lysate by about 25% at 30 *μ*M while it had little effect on SIRT1 level (Figures 5(a)–5(d)), indicating the specific effect of PL171 on SIRT3. Additionally, treatment with PL171 for 24 h significantly promoted mRNA level of *SIRT3* but not *SIRT1* (Figs. [Supplementary-material supplementary-material-1] and [Supplementary-material supplementary-material-1]). The time course experiments revealed that SIRT3 mRNA and protein expressions were sequentially increased in response to PL171 challenge, with the significant change present at 4 h for mRNA while 24 h for protein (Figs. [Supplementary-material supplementary-material-1]). The expression of the SIRT3 gene is shown to be controlled by the transcription factor PGC-1*α* which can be regulated by AMP-activated protein kinase (AMPK) signal pathway [31, 32]. Interestingly, the stimulation of PGC-1*α* mRNA and protein expressions by PL171 was detected (Figures 5(e) and 5(f) and Fig. [Supplementary-material supplementary-material-1]). Further, 30 *μ*M of PL171 stimulated AMPK phosphorylation and the pretreatment with AMPK inhibitor compound C (3 *μ*M, 30 min) abolished PL171-mediated AMPK activation and SIRT3 upregulation (Figures 5(g)–5(i)). All these suggest that PL171 promotes SIRT3 expression through AMPK/PGC-1*α* signal pathway.

Both SIRT3 and PGC-1*α* are declined in AD brain [17, 33]. Here, we found that A*β*_42_O (10 *μ*M, 24 h) reduced SIRT3 and PGC-1*α* expressions compared to the control group (Figure 5(j)). Pretreatment with PL171 for 4 h dose dependently attenuated A*β*_42_O-induced reduction of both SIRT3 and PGC-1*α* expressions (Figures 5(j)–5(l)). Notably, preincubation with 30 *μ*M of PL171 completely blocked the loss of SIRT3 and PGC-1*α* expressions regulated by A*β*_42_O.

### 3.6. PL171 Ameliorated A*β*_42_O-Induced Oxidative Stress and Mitochondrial Dysfunction via SIRT3

**Figure 6:**
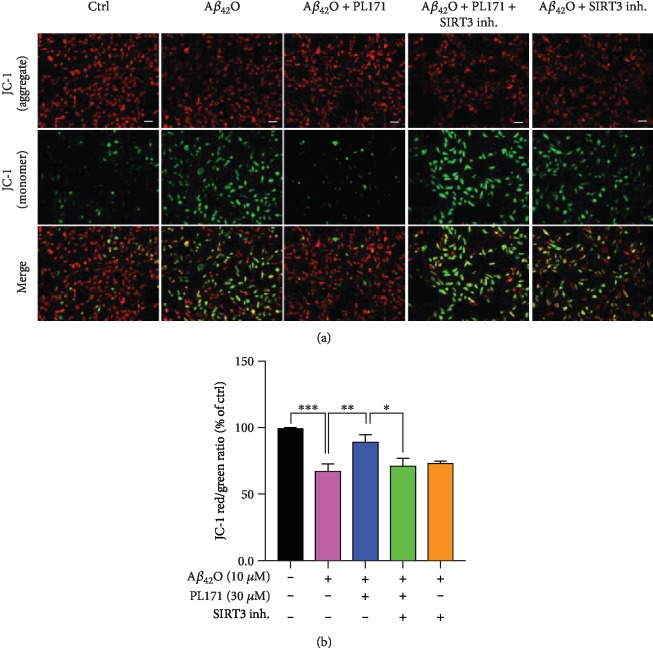
PL171 ameliorated A*β*_42_O-induced the impairment of mitochondrial dysfunction via SIRT3. (a) The representative image showing the MMP in SK-N-SH cells with indicated treatments. Scale bars, 50 *μ*m. (b) The quantification of relative MMP in (a). The data are presented as mean ± SEM, *n* ≥ 3 independent experiments, ^∗^*p* < 0.05, ^∗∗^*p* < 0.01, ^∗∗∗^*p* < 0.001, analyzed by one-way ANOVA followed by Bonferroni's test.

**Figure 7:**
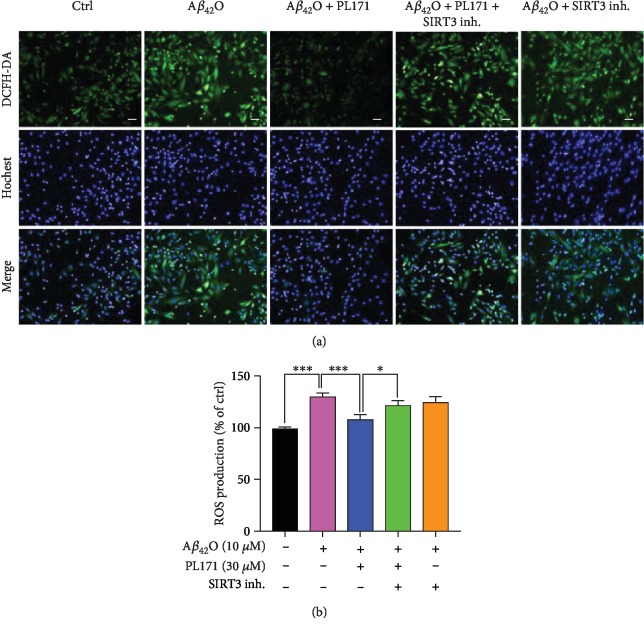
PL171 inhibited A*β*_42_O-promoted ROS production via SIRT3. (a) The image showing ROS levels in SK-N-SH cells after the indicated treatments. Scale bars, 50 *μ*m. (b) The quantification of relative ROS generation in (a). The data are presented as mean ± SEM, *n* ≥ 3 independent experiments, ^∗^*p* < 0.05, ^∗∗∗^*p* < 0.001, analyzed by one-way ANOVA followed by Bonferroni's test.

In order to verify whether the upregulation of SIRT3 is involved in the protective effect of PL171 on mitochondrial dysfunction caused by A*β*_42_O, we introduced a SIRT3 inhibitor, 3-TYP. A*β*_42_O (10 *μ*M, 24 h) reduced MMP by 32% to the control group, which was successfully prevented by PL171 (30 *μ*M, 4 h preincubation). In SIRT3 inhibitor-pretreated cells (20 *μ*M, 4 h), A*β*_42_O decreased MMP by 28% which was not changed by PL171 (Figures 6(a) and 6(b)). Meanwhile, PL171 inhibited A*β*_42_O-mediated increase of ROS level and this effect was attenuated when 3-TYP was applied with PL171 (Figures 7(a) and 7(b)). These results suggest that SIRT3 mediates the protective effects of PL171 on A*β*_42_O-induced oxidative stress and mitochondrial dysfunction.

### 3.7. PL171 Restored A*β*_42_O-Induced Cell Senescence through SIRT3 Modulation

**Figure 8:**
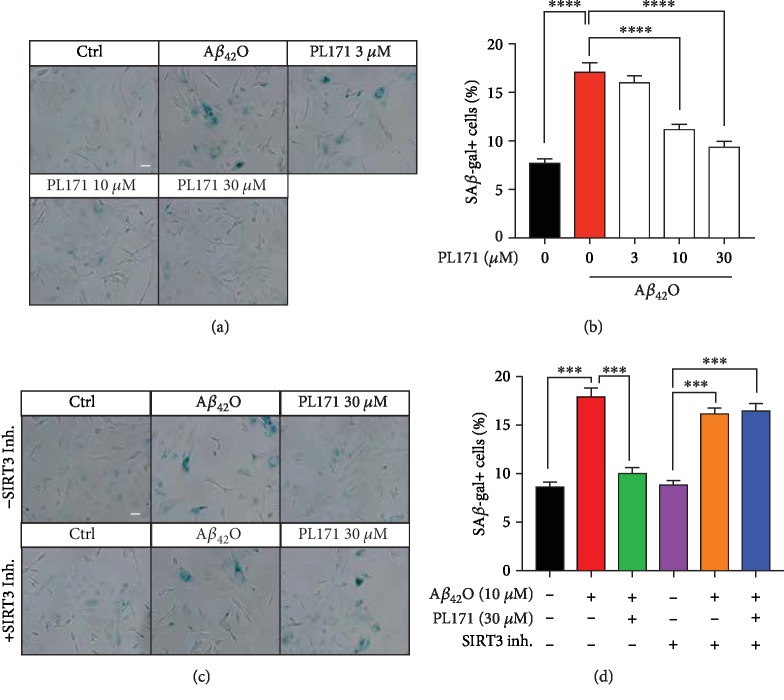
PL171 restored A*β*_42_O-induced cell senescence through SIRT3 modulation. (a) The representative images presenting SA-*β*-gal staining in the cells preincubated with PL171 (30 *μ*M) for 4 h followed by A*β*_42_O (10 *μ*M) challenge for 72 h. The images were obtained by a Zeiss Observer Z1 microscope. Scale bars, 50 *μ*m. (b) The quantification of relative number of SA-*β*-gal positive cells in (a). (c) The representative images of SK-N-SH cells treated as indicated for 72 h and stained for SA-*β*-gal. Scale bars, 50 *μ*m. (d) Quantification of relative number of SA-*β*-gal positive cells in (c). The data are presented as mean ± SEM, *n* ≥ 3 independent experiments, ^∗∗∗^*p* < 0.001 and ^∗∗∗∗^*p* < 0.0001, analyzed by one-way ANOVA followed by Bonferroni's test.

Mitochondrial dysfunction is related closely to cell senescence in neuronal cells. By staining of SA-*β*-gal, we observed that A*β*_42_O (10 *μ*M, 72 h) increased the number of SA-*β*-gal-positive cells by more than twofold (Figures 8(a) and 8(b)). This was attenuated by pretreatment with PL171 for 4 h in a dose-dependent manner. PL171 at 30 *μ*M reduced the number of A*β*_42_O-promoted SA-*β*-gal-positive cells to the level as the control. Moreover, in the cells with 20 *μ*M 3-TYP, A*β*_42_O (10 *μ*M, 72 h) resulted in a similar increase in the number of SA-*β*-gal-positive cells as in the cells without 3-TYP (Figures 8(c) and 8(d)). Cotreatment with PL171 and 3-TYP did not change the effect of A*β*_42_O. These indicate that PL171 protects neuronal cells from mitochondria-associated cell senescence induced by A*β*_42_O through promoting SIRT3 activity.

## 4. Discussion

In AD, mitochondrial dysfunction could be comprised of three different aspects: (1) mitochondrial dynamic or morphology, (2) bioenergetics (ATP and oxidative stress), and (3) transport [34]. Regarding bioenergetics defects of mitochondria in AD, many patients and disease models display reduced ATP production, excessive ROS generation, and significant respiratory defects [10]. Moreover, AD pathological proteins including A*β* and tau have been demonstrated to impair mitochondrial mass and function [35, 36]. Although it is unclear whether mitochondrial dysfunction comes earlier than the appearance of pathological proteins or not, all these studies emphasize the essential roles of mitochondria in AD pathogenesis and targeting mitochondria dysfunction could be beneficial for disease treatment. Indeed, a variety of antioxidants such as resveratrol [37, 38], curcumin [39], and idebenone have been shown to improve memory deficit in AD [40, 41]. In the present study, we demonstrate that a newly designed natural compound derivative PL171 may have antioxidant effects and prevent A*β*-induced mitochondrial dysfunction in human neuronal cells, indicating that PL171 could be a therapeutic agent for AD by targeting the mitochondria.

The mechanism of A*β*-mediated mitochondrial dysfunction is not exactly clear yet. In recent years, some groups have explored relevant mechanisms to impact mitochondrial function in AD. SIRT3 is the main mitochondrial sirtuin involved in protecting stress-induced mitochondrial integrity and energy metabolism and is highly associated with the pathogenesis of AD [13]. In the cortex of APP/PS1 double transgenic mice which are overproducing A*β*, both the mRNA and protein levels of SIRT3 are declined [42] and literature shows a negative association between SIRT3 expression and A*β* level in AD patients [17]. Thus, SIRT3 has been suggested as a molecular target for treating aging and age-related diseases [43, 44]. Here, we observed that A*β*_42_O induced the reduction of SIRT3 expression and its activity, further proving that SIRT3 is involved in A*β*-mediated mitochondrial dysfunction. The prevention of SIRT3 reduction by PL171 attenuated A*β*_42_O-induced neuronal defects, which were abolished by the SIRT3 inhibitor, suggesting that SIRT3 could be a therapeutic target for AD treatment.

PGC-1*α*, a transcriptional coactivator for the peroxisome proliferator-activated receptor-*γ* (PPAR*γ*) and for other transcription factors is involved in the regulation of oxidative phosphorylation, lipid metabolism, and mitochondrial biogenesis [45]. PGC-1*α* has protective effects against AD pathology. For example, PGC-1*α* has been reported to downregulate the transcription and expression of BACE1, which results in reduced A*β* generation and increased nonamyloidogenic sAPP*α* levels [46]. Notably, it was revealed that PGC-1*α* was decreased in the brain of AD patients and the content of PGC-1*α* protein was negatively correlated with A*β* levels [33]. Furthermore, *in vitro* studies demonstrated that A*β* reduced PGC-1*α* expression and PGC-1*α* could restore A*β* neurotoxicity [47, 48]. Here, PL171 significantly increased both mRNA and protein levels of PGC-1*α* and prevented A*β*-induced decline of protein. It has been reported that AMPK activation can stimulate CREB-mediated PGC-1*α* expression which regulates ERR*α* binding to the motif in SIRT3 promoter and promotes SIRT3 gene level [49, 50]. We observed that PL171 stimulated AMPK activation and its inhibition abolished the effect of PL171 on SIRT3. Thus, we suspect that PL171 may improve mitochondrial function via AMPK/PGC-1*α*/SIRT3 axis.

Cell senescence is a biological process that involves several key elements including mitochondrial dysfunction, ROS production, inflammation, and DNA damage and plays a key role in promoting aging and age-related diseases, such as AD [51, 52]. Together with previous studies [53, 54], our data show that long-term treatment with A*β*_42_O facilitate the number of senescent neuronal cells properly by stimulating ROS generation and mitochondrial dysfunction. Selective elimination of senescent cells or inhibition of cell senescence process is now considered a promising strategy for the treatment of age-associated disorders [55]. Rhamnose and rhamnoside have antioxidant effects and show benefits on skin aging [19, 20], while whether they could influence A*β*_42_O-induced neuronal senescence is unknown. Here, as a designed rhamnoside derivative, PL171 can not only prevent A*β*_42_O-induced oxidative stress and mitochondrial impairment but also inhibited A*β*_42_O-mediated cell senescence. All these effects were absent when the activity of SIRT3 was blocked, indicating that PL171 has antiaging or anti-AD effects via targeting SIRT3. Notably, all these effects of PL171 were examined in a cell line which is deficient for *in vivo* interoperation. In the future, more relevant *in vivo* models should be applied to further investigate the therapeutic potential of PL171 on aging or AD intervention.

## 5. Conclusion

PL171 can counteract A*β*-induced oxidative stress-mediated mitochondrial dysfunction and cell senescence via promoting SIRT3 function in human neuronal cells.

## Data Availability

All data used to support the findings of this study are included within the article.

## Supplementary Materials

Supplementary Materials Figure S1: the synthesis route for PL171. (A) Pyridine, CH2Cl2, 0°C to rt, 75% for *β*-L-rhamnopyranoside, and 11% for *α*-L-rhamnopyranoside. (B) TBAF, THF, rt; CH3NH2, MeOH, CH2Cl2, 0°C, 54% for two steps. Figure S2: the ^1^H-NMR spectrum of PL171. ^1^H-NMR (CD_3_OD, 400 MHz) *δ* 7.56 (d, *J* = 12 Hz, 1H) 7.19 (d, *J* = 4 Hz, 1H), 7.07 (dd, *J*_1_ = 6 Hz, *J*_2_ = 1 Hz, 1H), 6.82 (d, *J* = 4 Hz, 1H), 6.62 (d, *J* = 12 Hz, 1H), 5.28 (d, *J* = 1 Hz, C_1_-H), 3.92 (s, 3H),3.85 (d, *J* = 4 Hz, C_2_-H), 3.54-3.52 (m, C_3_-H), 3.38-3.34 (m, C_4/5_-H), 1.32 (d, *J* = 4 Hz, 3H). Figure S3: the ^1^H-^1^H COSY spectrum of PL171. ^1^H-NMR (CD_3_OD, 400 MHz) *δ* C_1_-H: 5.28, C_2_-H: 3.85, C_3_-H:3.54-3.52, C_4/5_-H:3.37-3.35 (m, 2H). Figure S4: the change of MMP in SK-N-SH cells incubated with A*β*_42_O at indicated concentrations (1 *μ*M, 3 *μ*M, and 10 *μ*M) and at indicated time (8 h, 16 h, and 24 h). The data are presented as mean ± SEM, ^∗∗∗^*p* < 0.001 and ^∗∗∗∗^*p* < 0.0001, and analyzed by one-way ANOVA followed by Bonferroni's test. Only the statistical analysis for 24 h was shown here, *n* ≥ 3 independent experiments. Figure S5: the dosage and time-course of PL171-mediated PGC-1*α*, SIRT1, or SIRT3. (A) SIRT3 mRNA levels were measured after treatment with PL171 at indicated concentrations for 24 h. (B) SIRT1 mRNA levels were measured after treatment with PL171 at indicated concentrations for 24 h. (C) PGC-1a mRNA levels were measured after treatment with PL171 at indicated concentrations for 24 h. (D) SIRT3 mRNA levels were measured after treatment with PL171(30 *μ*M) at indicated times. (E) Cells were treated with PL171(30 *μ*M) for indicated times, and cell lysates were prepared and analyzed using western blotting; the quantification of relative SIRT3 protein level was shown in (F). The data are presented as mean ± SEM, *n* ≥ 3 independent experiments, ^∗^*p* < 0.05 and ^∗∗^*p* < 0.01, and analyzed by one-way ANOVA followed by Bonferroni's test.
