# Supplementary material for: A Newly Synthesized Rhamnoside Derivative Alleviates Alzheimer's Amyloid-β-Induced Oxidative Stress, Mitochondrial Dysfunction, and Cell Senescence through Upregulating SIRT3
Source: Oxid Med Cell Longev. 2020 Feb 13;2020:7698560. doi: 10.1155/2020/7698560 (PMC7040408; doi:10.1155/2020/7698560)
Supplement: Supplementary Materials — Figure S1: the synthesis route for PL171. (A) Pyridine, CH2Cl2, 0°C to rt, 75% for β-L-rhamnopyranoside, and 11% for α-L-rhamnopyranoside. (B) TBAF, THF, rt; CH3NH2, MeOH, CH2Cl2, 0°C, 54% for two steps. Figure S2: the 1H-NMR spectrum of PL171. 1H-NMR (CD3OD, 400 MHz) δ 7.56 (d, J = 12 Hz, 1H) 7.19 (d, J = 4 Hz, 1H), 7.07 (dd, J1 = 6 Hz, J2 = 1 Hz, 1H), 6.82 (d, J = 4 Hz, 1H), 6.62 (d, J = 12 Hz, 1H), 5.28 (d, J = 1 Hz, C1-H), 3.92 (s, 3H),3.85 (d, J = 4 Hz, C2-H), 3.54-3.52 (m, C3-H), 3.38-3.34 (m, C4/5-H), 1.32 (d, J = 4 Hz, 3H). Figure S3: the 1H-1H COSY spectrum of PL171. 1H-NMR (CD3OD, 400 MHz) δ C1-H: 5.28, C2-H: 3.85, C3-H:3.54-3.52, C4/5-H:3.37-3.35 (m, 2H). Figure S4: the change of MMP in SK-N-SH cells incubated with Aβ42O at indicated concentrations (1 μM, 3 μM, and 10 μM) and at indicated time (8 h, 16 h, and 24 h). The data are presented as mean ± SEM, ∗∗∗p < 0.001 and ∗∗∗∗p < 0.0001, and analyzed by one-way ANOVA followed by Bonferroni's test. Only the statistical analysis for 24 h was shown here, n ≥ 3 independent experiments. Figure S5: the dosage and time-course of PL171-mediated PGC-1α, SIRT1, or SIRT3. (A) SIRT3 mRNA levels were measured after treatment with PL171 at indicated concentrations for 24 h. (B) SIRT1 mRNA levels were measured after treatment with PL171 at indicated concentrations for 24 h. (C) PGC-1a mRNA levels were measured after treatment with PL171 at indicated concentrations for 24 h. (D) SIRT3 mRNA levels were measured after treatment with PL171(30 μM) at indicated times. (E) Cells were treated with PL171(30 μM) for indicated times, and cell lysates were prepared and analyzed using western blotting; the quantification of relative SIRT3 protein level was shown in (F). The data are presented as mean ± SEM, n ≥ 3 independent experiments, ∗p < 0.05 and ∗∗p < 0.01, and analyzed by one-way ANOVA followed by Bonferroni's test. [file 7698560.f1.zip › Supplementary Figure Legends.docx]

**Supplementary Figure Legends**

Fig.S1 The synthesis route for PL171

(A) Pyridine, CH2Cl2, 0 ^o^C to rt, 75% for β-L-rhamnopyranoside, and 11% for α-L-rhamnopyranoside

(B) TBAF, THF, rt; CH3NH2, MeOH, CH2Cl2, 0 oC, 54% for two steps

Fig.S2 The ^1^H-NMR spectrum of PL171.

^1^H-NMR (CD_3_OD, 400 MHz) δ 7.56 (d, J = 12 Hz, 1H) 7.19 (d, J = 4Hz, 1H), 7.07 (dd, J_1_ = 6 Hz, J_2_ = 1 Hz, 1H), 6.82 (d, J = 4 Hz, 1H), 6.62 (d, J = 12 Hz, 1H), 5.28 (d, J = 1 Hz, C_1_-H), 3.92 (s, 3H),3.85 (d, J = 4 Hz, C_2_-H), 3.54-3.52 (m, C_3_-H), 3.38-3.34 (m, C_4/5_-H), 1.32 (d, J = 4 Hz, 3H).

Fig.S3 The ^1^H-^1^H COSY spectrum of PL171.

^1^H-NMR (CD_3_OD, 400 MHz) δ C_1_-H: 5.28, C_2_-H: 3.85, C_3_-H:3.54-3.52, C_4/5_-H:3.37-3.35 (m, 2H).

Fig.S4 The change of MMP in SK-N-SH cells incubated with Aβ_42_O at indicated concentrations (1μM, 3μM, 10μM) and at indicated time (8h, 16h, 24h). The Data are presented as mean ± SEM, ***p < 0.001, and ****p < 0.0001, analyzed by one-way ANOVA followed by Bonferroni test. Only the statistical analysis for 24 h was shown here, n ≥ 3 independent experiments.

Fig.S5 The dosage and time-course of PL171-mediated PGC-1α, SIRT1, or SIRT3.

(A) SIRT3 mRNA levels were measured after treatment with PL171 at indicated concentrations for 24h.

(B) SIRT1 mRNA levels were measured after treatment with PL171 at indicated concentrations for 24h.

(C) PGC-1a mRNA levels were measured after treatment with PL171 at indicated concentrations for 24h.

(D) SIRT3 mRNA levels were measured after treatment with PL171(30 μM) at indicated times.

(E) Cells were treated with PL171(30 μM) for indicated times, and cell lysates were prepared and analyzed using western blotting; the quantification of relative SIRT3 protein level was shown in (F).

The Data are presented as mean ± SEM, n ≥ 3 independent experiments, *p < 0.05, **p < 0.01, analyzed by one-way ANOVA followed by Bonferroni test.
